# Alpha-glucosidase and amylase inhibitory effects of *Eruca vesicaria* subsp. *longirostris* essential oils: synthesis of new 1,2,4-triazole-thiol derivatives and 1,3,4-thiadiazole with potential inhibitory activity

**DOI:** 10.1080/13880209.2019.1642363

**Published:** 2019-08-27

**Authors:** Fayçal Hichri, Amel Omri, Aisha Saad Mana Hossan, Hichem Ben Jannet

**Affiliations:** a Chemistry Department, Faculty of Science, King Khaled University, Abha, Saudi Arabia;; b Laboratoire de Chimie hétérocyclique, Produits Naturels et Réactivité, Equipe: Chimie Médicinale et Produits Naturels, Faculté des Sciences de Monastir, Université de Monastir, Monastir, Tunisia;; c Faculté de Pharmacie, Laboratoire des maladies transmissibles et des substances biologiquement actives, Monastir, Tunisie

**Keywords:** Erucin, natural precursor, hypoglycemic

## Abstract

**Context:** The substantial increase in the number of diabetics has encouraged the search for new pharmacological strategies to face this problem. In this regard, triazole and its derivatives have attracted considerable attention for the past few decades due to their pharmacological significance.

**Objective:** Evaluation of the inhibitory activity of α-glucosidase and α-amylase in essential oils extracted from plant *Eruca vesicaria* (L) Cav. subsp. *longirostris* (Brassicaceae) (EVL) and to verify whether the triazoles and thiadiazol bearing the lipophilic 4-methylthiobutyl group synthesized from the essential oil contribute to this activity.

**Materials and methods:** The essential oils were extracted by hydrodistillation from leaf, stem, root, and fruit of EVL, and their chemical compositions were analyzed by gas chromatography and gas chromatography-mass spectrometry. We present here the synthesis of three new types of 1,2,4-triazole-thiol and 1,3,4-thiadiazol and the structures were confirmed by NMR, mass spectrometry. The α-glucosidase and α-amylase inhibitory activities were investigated *in vitro*.

**Results:** The main compound in fruit, stem, and root was erucin (96.6, 85.3, and 83.7%, respectively). The three essential oils of the fruit, stem, and root have strong inhibitory activity on α-glucosidase and α-amylase; IC_50_ values of roots were 0.81 ± 0.02 μg/mL and 0.13 ± 0.01 μg/mL, respectively. Derivatives **1 b**, **2 b**, **3 b,** and **2c** showed remarkable inhibitory activity against α-glucosidase with potencies better than that of acarbose with IC_50_ values ranging between 0.49 and 1.43 μM.

**Conclusions:** Current results indicate that ECL fruit essential oil can be used as a natural precursor for the synthesis of triazoles as potential hypoglycemic agents.

## Introduction

Diabetes is a growing health problem in the world and is becoming an epidemic around the world. Diabetes mellitus (DM) is the most common endocrine disease worldwide. According to reports published by World Health Organization (WHO) in 2016; about 173 million people suffer from diabetes mellitus, and this number is expected to reach 366 million by 2030.

Plants are a huge bank of chemicals from which we can explore potential therapeutic agents by bioactivity-targeted screening. Screening of plants inhibiting α-glucosidase is increasing (Gholamhoseinan et al. 2008). About 800 plant species have been reported to have antidiabetic properties. The cruciferous family, Brassicaceae, is an economically important family as it includes many food and oilseed crops. It is a large family comprising 3700 species spread over 338 genera (Simpson 2010). The family members are distinguished by having a pungent odour and sulphur due to volatile isothiocyanate derivatives, obtained upon hydrolysis of glucosinolates (Lin et al. 2000; Afsharypuor and Hoseiny-Balam 2005). Some glucosinolate degradation products are used in organic synthesis (Abbott et al. 2002). However, the isolation of these isothiocyanates requires large amounts of plant material and requires tedious extraction procedures, sometimes with multiple purification steps, generating only a low yield. In our present study, the essential oils of *Eruca vesicaria* (L) Cav. subsp. *longirostris* (Brassicaceae) (EVL) are used as a source of erucin (4-methylthiobutyl isothiocyanate), employed as a natural precursor for the simple synthesis of new α-glucosidases inhibitors of triazole. Heterocycles containing triazole ring systems are well known to possess a variety of biological activities, including anti-inflammatory (Amir et al. 2008), antiviral (Balba et al. 2011), antitumor (Balba et al. 2011) and especially antidiabetic (Demirbaş et al. 2002; Jabeen et al. 2014). A set of triazole compounds synthesized by Jabeen et al. (2014) was based on a series of analyses of ‘pre-screening’ QSAR (Quantitative Structure-Activity Relationship) and MFTA (Molecular Field Analysis Topology) exhibiting α-glucosidase inhibitory activity. This previous report suggested that the presence of a lipophilic side chain in the molecule is available for the α-glucosidase inhibitory activity Jabeen et al. (2014). Recently, two new 1,2,3-triazoles containing a lipophilic moiety have been isolated from the roots of *Paramignya trimera* (Oliv.) Guillaum (Satya et al. 2018). These roots have been used as traditional Vietnamese medicines for the treatment of diabetes and their α-glucosidase inhibitory activities have been examined (Satya et al. 2018).

To the best of our knowledge and according to literature survey, there are no reports on the alpha-glucosidase and amylase inhibitory effects of essential oils from EVL and this is the first report for the synthesis of 1,2,4-triazole-thiol derivatives and 1,3,4-thiadiazol bearing the 4-methylthiobutyl as lipophilic part together with their α-glucosidase inhibitory activity, by employing erucin from the essential oil of fruits as a natural precursor of these syntheses.

## Materials and methods

### Collection and extraction of essential oils

EVL was collected at the flowering stage in February 2012 from the area of Kasserin-Tunisia. The botanical identification was carried out by Dr Fethia Harsallah-Skhiri, botanist in High Institute of Biotechnology of Monastir, Tunisia. A voucher specimen was deposited at the Laboratory of Medicinal Chemistry and Natural Products at the Faculty of Sciences of Monastir, Tunisia. The flowers, leaves, stems and roots were divided into small pieces and weighed before the extraction of the volatile compounds.

Extraction was carried out by hydrodistillation for 4 h, using a Clevenger-type apparatus. The essential oil was collected by decantation, then dried over anhydrous sodium sulphate, weighed and stored in sealed glass vials at 4-5 °C until analysis. Yield based on the fresh weight of the sample was calculated.

### Chemistry

#### General procedure for the synthesis of thiourea derivatives

The thiourea derivatives were synthesized by a known method (Guda et al. 2012). The experimental procedure was typical for synthesis of 1-(4-(methylthiobutyl)-3-phenylthiourea: a mixture of aniline (2 mmol), erucin of the essential oil of fruits (2 mmol) and ethanol (2 mL) were refluxed for 2–5 h. After evaporation of the solvent, the crude product was diluted in distilled water, washed with a solution of HCl (3 M) and then extracted with ethyl acetate. The organic layer was dried by anhydrous Na_2_SO_4_. After the evaporation of the solvent, the crude product was purified by silica gel column chromatography (hexane/ethyl acetate 7:3). All obtained compounds were new.

*2-Acetyl-N-(4-methylthiobutyl) hydrazinecarbothioamide* **(1a):** white powder; ^1 ^H NMR (300 MHz, CDCl_3_): *δ* 3.99 (t, 2 H, *J* 7.2 Hz), 2.54 (t, 2 H, *J* 7.2 Hz), 2.39 (s, 3 H), 2.08 (s, 3 H), 1.84 (m, 2 H), 1.67 (m, 2 H); ^13 ^C NMR (CDCl_3_): *δ* 184.1, 168.3, 43.2, 33.0, 26.8, 15.0, 11.0 ppm; ESIMS *m/z*: calcd C_8_H_17_N_3_OS_2_ [M + H]^+^ 236.0, found 236.0.

*2-Benzoyl-N-(4-methylthiobutyl) hydrazinecarbothioamide* **(2a):** white powder; ^1 ^H NMR (300 MHz, CDCl_3_): *δ* 7.73 (d, 2 H, *J* 7.5 Hz), 7.39 (t, 1 H, *J* 7.5 Hz), 7.30 (d, 2 H, *J* 7.5 Hz), 3.42 (t, 2 H, *J* 6.6 Hz), 2.31 (t, 2 H, *J* 7.2 Hz), 2.07 (s, 3 H), 1.70 (m, 4 H); ^13 ^C NMR (CDCl_3_): *δ* 182.2, 167.6, 131.6, 127.6, 126.9, 124.7, 43.1, 32.7, 27.3, 25.4, 15.3 ppm; ESIMS *m/z:* calcd C_13_H_19_N_3_OS_2_ [M + H]^+^ 298.1, found 298.1.

*N-(4-Methylthiobutyl)-2-(thiophene-2-carbonyl)hydrazinecarbothioamide* **(3a):** White powder; ^1 ^H NMR (300 MHz, MeOD): *δ* 7.80 (d, 1 H, *J* 3.9 Hz), 7.73 (d, 1 H, *J* 3.9 Hz), 7.16 (t, 1 H, *J* 3.9 Hz), 3.59 (t, 2 H, *J* 6.6 Hz), 2.50 (t, 2 H, *J* 7.2 Hz), 2.05 (s, 3 H), 1.67 (m, 4 H); ^13 ^C NMR (MeOD): *δ* 184.1, 132.6, 130.8, 130.1, 128.9, 45.1, 34.7, 29.3, 27.3, 15.3 ppm; ESIMS *m/z:* calcd C_11_H_17_N_3_OS_3_ [M + H]^+^ 304.0, found 304.0.

#### General method for the synthesis of compounds **1 b, 2 b and 3 b**

The resulting thiourea was mixed with a solution of KOH (4 mL, 2 N). The mixture was kept under reflux and the evolution of the reaction was monitored by TLC. At the end of the reaction, the mixture was neutralized with an acetic acid solution to pH∼6 and then diluted with distilled water and extracted with AcOEt. The obtained organic phase was dried over anhydrous Na_2_SO_4_. After evaporating the solvent, the resulting residue was washed with ethanol, then purified on a silica gel column eluted with the mixture Cyclohexane/AcOEt (7: 3) to give the corresponding triazoles: **1 b**, **2 b** and **3 b**. The yields of the obtained products were between 66 and 76%.

*5-Methyl-4-(4-methylthiobutyl)-4H-1,2,4-triazole-3-thiol* **(1 b):** White powder; Rd = 72%, mp = 92 °C; 1 H NMR (CDCl_3_, 300 MHz): δ (ppm) = 1.69 (q, 2 H, H-6, *J* = 7.2 Hz); 1.85 (q, 2 H, H-5, *J* = 7.2 Hz); 2.07 (s, 3 H, H-8); 2.39 (s, 3 H, H-1); 2.51 (t, 2 H, H-7, *J* = 7.2 Hz); 3.98 (t, 2 H, H-4, *J* = 7.5 Hz); 13 C-NMR (CDCl_3_, 75 MHz) δ (ppm) = 11.5 (C-1); 15.4 (C-8); 25.9 (C-6); 27.1 (C-5); 33.5 (C-7); 43.7 (C-4); 151.0 (C-5); 162.3 (C-3); ESIMS *m/z:* [M + H]^+^ 218.0.

*4-(4-Methylthiobutyl)-5-phenyl-4H-1,2,4-triazole-3-thiol* **(2 b):** White powder; Rd = 76%, mp = 101 °C; 1 H NMR (CDCl 3, 300 MHz): δ (ppm) = 1.56 (q, 2 H, H-11, *J* = 7.2 Hz); 1.83 (q, 2 H, H-10, *J* = 7.2 Hz); 2.02 (s, 3 H, H-13); 2.42 (t, 2 H, H-12, *J* = 7.2); 4.11 (t, 2 H, H-9, *J* = 7.5 Hz); 7.56 (m, 5 H, H-aromatics); 12.22 (s, 1 H, SH); 13 C-NMR (CDCl_3_, 75 MHz) δ (ppm) = 14.8 (C-13); 25.3 (C-11); 26.7 (C-10); 32.8 (C-12); 43.8 (C-9); 125.4 (C-2 and C-6); 128.1 (C-3 and C-5); 128.7 (C-1), 130.6 (C-4), 151.6 (C-7), 167.2 (C-8); ESIMS *m/z:* [M + H]^+^ 280.1.

*4-(4-Methylthiobutyl)-5-(thiophen-2-yl)-4H-1,2,4-triazole-3-thiol* **(3 b):** White powder; Rd = 66%, mp = 104 °C; 1 H NMR (CDCl 3, 300 MHz): δ (ppm) = 1.69 (q, 2 H, H-6, *J* = 7.5 Hz); 1.85 (q, 2 H, H-5, *J* = 8.1 Hz); 2.07 (s, 3 H, H-8); 2.39 (s, 3 H, H-1); 2.51 (t, 2 H, H-7, *J* = 7.2 Hz); 3.98 (t, 2 H, H-4, *J* = 7.8 Hz); 13 C-NMR (CDCl_3_, 75 MHz): δ (ppm) = 11.5 (C-1); 15.4 (C-8); 25.9 (C-6); 27.1 (C-5); 33.5 (C-7); 43.7 (C-4), 128.1 (C-8); 129.1 (C-9); 129.3 (C-7); 141.1 (C-6); 154.2 (C-5); 162.6 (C-3); ESIMS *m/z:* [M + H]^+^ 286.0.

#### General method for the synthesis of compounds 2c

The resulting thiourea was mixed with concentrated H_2_SO_4_ solution (5 mL) in an ice bath. The mixture was stirred at room temperature and the progress of the reaction was monitored by TLC. At the end of the reaction, the mixture was neutralized with an ammonia solution to pH 8, diluted with distilled water and extracted with CHCl_3_.The obtained organic phase was dried over anhydrous Na_2_SO_4_. After evaporating the solvent, the resulting residue was washed with ethanol, then purified on a silica gel column eluted with the mixture Cyclohexane/AcOEt (7: 3) to give the corresponding thiapirazole.

*N-(4-Methylthiobutyl)-5-phenyl-1,3,4-thiadiazol-2-amine* **(2c):** White powder; Rd = 83%, mp 94 °C; 1 H NMR (CDCl_3_, 300 MHz): δ (ppm) = 1.75 (m, 2 H, H-11); 1.86 (m, 2 H, H-10); 2.10 (s, 3 H, H-13); 2.57 (t, 2 H, H-12, *J* = 7.2 Hz); 3.42 (t, 2 H, H-9, *J* = 6.9 Hz); 7.44 (m, 5 H, aromatic H,); 13 C-NMR (CDCl_3_, 75 MHz) δ (ppm) = 15.0 (C-13); 25.8 (C-11); 27.8 (C-10); 33.3 (C-12); 46.5 (C-9); 126.3 (C-2 and C-4); 128.4 (C-1 and C-5); 129.2 (C-3); 156.8 (C-8); 169.9 (C-7); ESIMS *m/z:* [M + H]^+^ 280.1.

#### Determination of α-Glucosidase inhibition activity

α-Glucosidase inhibitory activity was determined as previously described by Tao et al. (2013), with some modifications as detailed by Rengasamy et al. (2013). Yeast α-glucosidase (Cat. No. G 5003, Sigma Aldrich Chemical Co, USA) reaction mixture contained 2.5 mM *p*-nitrophenyl-α-d-glucopyranoside (pNPG), 250 µL of products (the concentrations were varied from 1-1000 μM) in DMSO and 0.3 U/mL of α-glucosidase in phosphate buffer, pH 6.9. Control tubes contained only DMSO, enzyme and substrate, while in positive controls acarbose replaced the solution of the product. Absorbance of the resulting *p*-nitrophenol (pNP) was determined at 405 nm and was considered directly proportional to the activity of the enzyme. The products were tested for α-glucosidase inhibitory activity at different concentrations (100-0.015 µg/mL). Each sample creation was performed in triplicate.

Inhibition Percentage by products and acarbose were calculated using the following equation:

$$\text{Inhibition}\;\text{Percentage}\left(\%\right)=1–\left(\left(\Delta {\text{OD}}_{\text{sample}}/\Delta {\text{OD}}_{\text{control}}\right)\times 100\right).$$

The IC_50_, which is the concentration of the sample required to inhibit 50% of the enzyme, was determined for each sample. All products were compared on the basis of their IC_50_ values estimated from the dose-response curves.

Different concentrations of plant extracts ranging from 10 μg/mL to 100 μg/mL.

#### Determination of α-amylase inhibition activity

The α-amylase inhibition activity of various EVL essential oils was determined according to the assay described by Worthington (1993). Different concentrations of each essential oil ranging from 10 to 100 μg/mL were prepared in DMSO. Essential oil (250 μL) of each concentration and 250 μL of α-amylase isolated from *Aspergillus oryzae* (Sigma-Aldrich) (0.1 U/mL) were taken and incubated at 25 °C for 10 min. After the pre-incubation, 250 µL of 1% starch solution in 0.02 M sodium phosphate buffer (pH 6.9) was added to each tube at regular time intervals. After the incubation at 25 °C for 30 min, the reaction was stopped with 0.1 mL of dinitrosalicylic acid reagent. The test tubes were then incubated in a boiling water bath for 5 min and cooled to room temperature. The reaction mixture was then diluted with the addition of 5 mL of distilled water and the absorbance was measured at 540 nm. The readings were compared with the control, which contains DMSO and buffer instead of sample extract. In the positive test, the acarbose replaces the extract. The % inhibition was calculated by using the following formula:

$$\alpha -\text{Amylase}\;\text{inhibition}\;\text{activity}\left(\%\right)=1-(\Delta {\text{DO}}_{\text{extract}}/\Delta {\text{DO}}_{\text{control}})\times 1$$

## Results and discussion

### Chemical composition of the essential oils

The essential oils of EVL were obtained by steam distillation with yields ranges from 0.003% (stems) to 0.018% (fruit). Table 1 shows the identified chemical constituents, their linear retention indices, and percentage compositions, listed in order of elution in the HP-5 capillary column. A total of 26 components were characterized using GC/MS analyses. The compound classes represented in these oils were sesquiterpene hydrocarbons, oxygenated sesquiterpenes, sulphur and/or nitrogen compounds, apocarotenoids, phenylpropanoids and other compounds. Most of the chemical compositions present in the three essential oils of fruits, stems and roots are isothiocyanate, the highest being the 4-methylthiobutyl isothiocyanate, also known as erucin (96.6, 85.3 and 83.7%, respectively), followed by 5-(methylthio)-pentanenitrile (2.3, 6.8 and 13.5%, respectively). However, the composition of the essential oil of the leaves is very different: β-elemene (35.7%), followed by hexahydrofarnesylacetone (23.9%), (*E*)-β-damascone (15.4%), erucin (10.6%) and α-longipinene (9.6%). Many studies have shown that β-elemene mark the characteristics of a broad spectrum of activities. Previous findings have also revealed that the inhibition of α-glucosidase and α-amylase can be attributed to the presence of monotepernes and sesquiterpenes in the essential oils (Obo et al. 2015; Adefegha et al. 2017). Qualitatively, the chemical composition of the essential oils of roots, stems and fruits suggest the possibility of using them as a source of erucin.

**Table 1.** Chemical composition of the essential oils isolated from the roots, stems, leaves and flowers of *E. longirostris*.

LRI	Compound	Roots (%)	Stems (%)	Leaves (%)	Fruits (%)
949	5-methyl-hexanenitrile	0.2			0.1
985	heptanenitrile	0.1			
1006	1-(methylthio)-hexane	0.1			
1043	β-isophorone				0.1
1099	pentyl isothiocyanate	0.1			
1120	isophorone				0.1
1165	4-methylpentyl isothiocyanate	0.5	1.2		
1193	3-hydroxy-2-ethyl-γ-pyrone (=2-ethylpyromeconic acid)	0.2	0.6		0.1
1202	5-(methylthio)-pentanenitrile	13.5	6.8		2.3
1285	*(E)*-anethole	0.1			
1312	3-methylthiopropyl isothiocyanate	0.2	0.4		
1314	4-vinyl guaiacol				0.1
1320	4-hydroxy-3-methylacetophenone	0.1			
1351	α-longipinene			9.6	
1383	*(E)*-β-damascenone				0.1
1392	β –elemene			35.7	
1412	*(E)*- β –damascone			15.4	
1431	erucin (=4-methylthiobutyl isothiocyanate)	83.7	85.3	10.6	96.6
1486	*(E)*-b-ionone			2.7	0.1
1562	ledene alcohol	0.1	1.4		
1582	caryophyllene oxide		0.5		
1716	pentadecanal	0.1			
1765	tetradecanoic acid	0.1	0.3		
1781	1-pentadecanol	0.1	0.3		
1817	hexadecanal		0.3		
1843	hexahydrofarnesylacetone		0.2	23.9	0.1
	sesquiterpene hydrocarbons	0.0	0.0	45.3	0.0
	oxygenated sesquiterpenes	0.1	1.9	0.0	0.0
	apocarotenoids	0.0	0.2	42.0	0.5
	sulphur and/or nitrogen compounds	98.4	93.7	10.6	99.0
	phenylpropanoids	0.1	0.0	0.0	0.0
	non-terpene derivatives	0.6	1.5	0.0	0.2
	total identified	99.2	97.3	97.9	99.7
	yield (%)	0.009	0.003	0.016	0.018

Note: LRI: linear retention index determined on the polar column DB-5 rel. to a series of *n*-alkane.

### Inhibitory activity test of the essential oils

The inhibitory activity of EVL essential oils on α-amylase and α-glucosidase was investigated in this study and the results are shown in Table 2. α-Amylase and α-glucosidase are the well-known enzymes playing a key role in the management of hyperglycaemia-linked type 2 diabetes (Bailey 2003).

**Table 2.** α-Glucosidase and α-amylase inhibition activity of essential oils of *E. longirostris*.

Extracts	CI_50_ (μg/mL) α-glucosidase inhibition	CI_50_ (μg/mL) α-amylase inhibition
Leaves	100 ± 3	21.20 ± 0.50
Fruits	1.12 ± 0.05	0.20 ± 0.02
Stems	0.87 ± 0.02	0.17 ± 0.01
Roots	0.81 ± 0.02	0.13 ± 0.01
Acarbose	280 ± 10	80.34 ± 1.00

As shown in Table 2, the effectiveness of the glucosidase and amylase inhibitors of the various EVL essential oils was compared on the basis of their IC_50_ values. High values of IC_50_ indicate low inhibitory activity. The three essential oils of fruits, stems and roots have strong inhibitory activity on α-glucosidase. Their IC_50_ values were 0.81 ± 0.02 μg/mL (roots), 0.87 ± 0.02 μg/mL (stems) and 1.12 ± 0.05 μg/mL (fruits), while the IC_50_ value of the positive control acarbose was 280 ± 10 μg/mL. The erucin could be responsible for the inhibition of α-glucosidase, as it is present at high levels in the active samples (83.7, 85.3, and 96.6%, respectively). In addition, this compound is present at low amount in the essential oil of the leaves (10.6%). Although, this essential oil has demonstrated a significant inhibition with the IC_50_ value 100 ± 3 μg/mL. The β-elemene content was 35.7%, suggesting that this sesquiterpene could contribute to the α- glucosidase inhibitory activity. It was reported that administration of terpenes to diabetic exerts blood glucose lowering effect in alloxan-induced diabetic rat (Hamden et al. 2011). Moreover, all samples have stronger α-amylase inhibition than α-glucosidase. The three essential oils of fruits, stems and roots exhibited strong inhibitory activity against α-amylase with IC_50_ values of 0.20 ± 0.02, 0.17 ± 0.01 and 0.13 ± 0.01 µg/mL, respectively. All mentioned oils showed stronger inhibition activity in comparison with the reference drug, acarbose (IC_50_ = 80.34 µg/mL). For the first look, one would expect that the high activity of these oils is due mainly to its dominant component i.e., 4-methylthiobutyl isothiocyanate (erucin). However, the fruit oil, while containing much lesser amount of that compound (Table 1) yet its activity is significant; it exhibited moderate α-amylase inhibition potency with IC_50_ of 21.20 ± 0.50 μg/mL.

### Chemistry

**Scheme 1.**
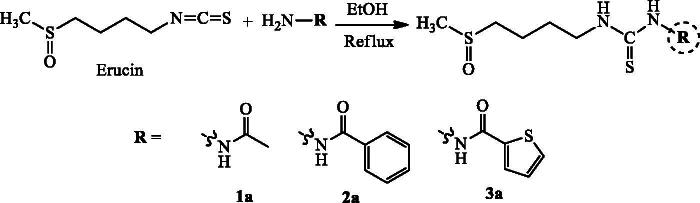
Synthesis of thioureas.

**Scheme 2.**
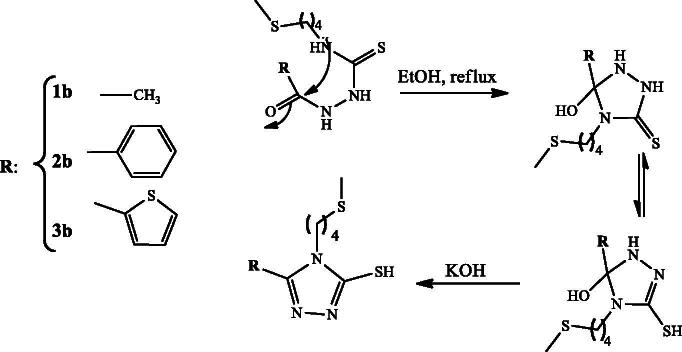
Synthesis of the 4-(4-(methylthio)butyl)-4H-1,2,4-triazole-3-thiols **1 b**, **2 b** and **3 b**.

**Scheme 3.**
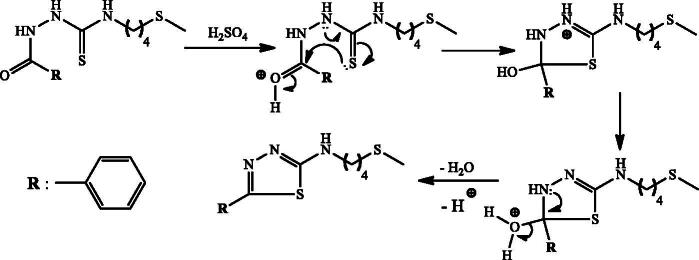
Synthesis of the *N*-(4-(methylthio)butyl)-5-phenyl-1,3,4-thiadiazol-2-amine **2c**.

In order to explore new anti-diabetic agents with drug-like properties, we herein report the synthesis of 1,2,4-triazole-thiol and 1,3,4-thiadiazol with their inhibitory activity against α-glucosidase. Several synthetic protocols of mercapto-1,2,4-triazoles have been reported previously (Kap-Sun et al. 2005; Bibian et al. 2010; Lässig et al. 2010). The condensation reaction of relatively small and linear molecules with suitable reagents is a general method leading to the formation of heterocyclic systems (Pesson et al. 1962). For this purpose, thioureas which incorporate hydrazide function in their structures, is a suitable precursor for the synthesis of triazoles and thiapirazoles. Indeed, we report here a two-step protocol for this synthesis starting from the natural precursor, which is the essential oil of the fruits containing erucin (96.6%). The first step involves the synthesis of different thiourea incorporating a hydrazide function in structures **1a**, **2a** and **3a** (Scheme 1). The second stage involved the condensation reaction of the synthesized thiourea under basic catalysis as reported by Balba et al. (2011) to obtain mercapto-1,2,4-triazoles **1 b**, **2 b** and **3 b** (Scheme 2), and under acid catalysis as reported by Guda et al. (2012) to give thiapirazole **2c** (Scheme 3). Their structures were characterized by MS, ^1 ^H NMR and ^13 ^C NMR spectra. ESIMS of all the derivatives were also in agreement with their molecular formula.

The plausible mechanism for the formation of mercapto-1,2,4-triazoles is shown in Scheme 2; the addition of base allows the cyclization of the prepared thiourea via attack of the nitrogen doublet on the carbonyl and then the dehydration allowing access to the triazoles **1 b**, **2 b** and **3 b**. Scheme 3 describes the synthesis of compounds **2c**. Briefly, the protonation of the carbonyl function of the prepared thiourea amide, followed by removal of a water molecule, allows the production of thiapirazole **2c**.

### α-Glucosidase inhibition of compounds 1 b, 2 b, 3 b and 2c

All compounds **1 b**, **2 b**, **3 b** and **2c** have been found to be highly effective inhibitor of α-glucosidase, exhibiting very potent inhibitory activity, with IC_50_ values ranging between 0.49 and 1.43 μM (Table 3). The strong enzyme-inhibitory activity was shown by compound **2 b** (IC_50_ = 0.49 μM). In contrast, the commercial inhibitor, acarbose, exhibited α-glucosidase inhibitory activity with an IC_50_ value equal to 108.8 ± 12 μM.

**Table 3.** IC_50_ values from compounds **1 b**, **2 b**, **3 b** and **2c** against α- glucosidase.

Compound	Structure	IC_50_ (μM)
**1b**	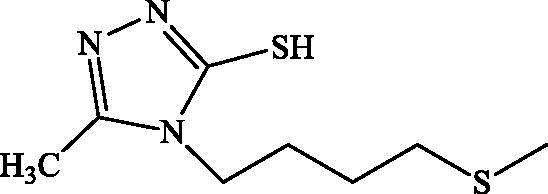	1.42 ± 0.80
**2b**	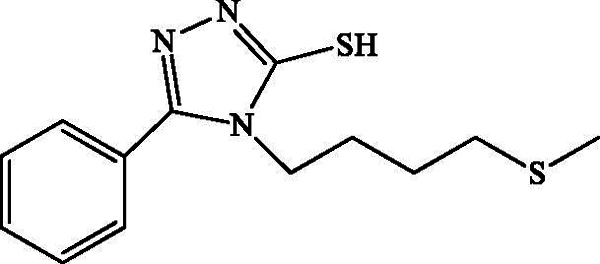	0.57 ± 0.05
**3b**	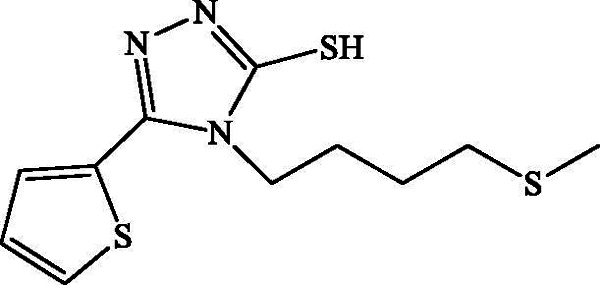	0.49 ± 0.03
**2c**	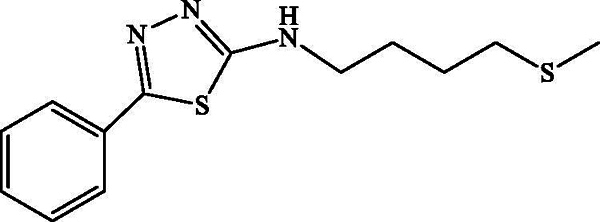	1.43 ± 0.09
Acarbose		108.80 ± 12.00

The synthesized analogues **1 b**, **2 b**, **3 b** and **2c** possess the specific structural characteristics responsible for α-glucosidase activity, identified in previous studies (Jabeen et al. 2014). It should be noted that, in the three different hydrazides selected for the synthesis of compounds **1 b**, **2 b** and **3 b**, the presence of the 4-methylthiobutyl lipophilic group retains an inhibitory activity and the IC_50_ values range from 0.49 to 1.42 μM. Meanwhile, the modification of the triazole ring by the thiadiazole ring did not significantly change the activity, and it remains important. For this reason, we believe that the presence of the 4-methylthiobutyl chain is playing a significant role in terms of lipophilicity and in promoting inhibitory activity for the synthesized derivatives. It can also be concluded that the presence of the sulphur atom contributes to this activity.

## Conclusions

In summary, the search for α-glucosidase inhibitors, derived from triazole, is important because they can potentially suppress postprandial hyperglycaemia in diabetic patients. The present work reported for the first time the α-glucosidase and α-amylase inhibitory effect of EVL essential oils. It is important to note that this essential oil and these derivatives of erucin strongly inhibited α-glucosidase in this study. Although it is not clear whether our new compounds, 1,2,4-triazole-thiol or 1,3,4-thiadiazol, and the EVL essential oils via α-glucosidase inhibition could suppress hyperglycaemia, elucidation of the inhibition mechanism is now under investigation. Furthermore, oils and synthesized compounds should be subjected to suitable *in vivo* experiments in order to assess and evaluate their antidiabetic potential. The next step will be to attempt to expand the range of these compounds to further confirm these results.
